# Construction Notes

**Published:** 1893-12-09

**Authors:** 


### CONSTRUCTION NOTES.

**New Infirmary at Guisborough Workhouse.**

This infirmary will contain accommodation for males and females; in all forty-two beds. In the centre of the building is arranged the nurses' apartments, ward sculleries, stairs, entrance, &c., with the female wards on one side, the male on the other. On the ground floor for each sex there is a day-room and a ward. Above these there are two large wards, accommodating twenty-two beds. Each ward will be provided with bath-room, lavatory, &c. The heating of the wards will be effected by Shoreland's patent combination open fireplace Manchester stoves. At each end of the building will be fixed cast-iron stairs, to be used in case of fire. The contract for the entire works amounts to £3,200. Mr. Thomas Stokes, architect, of Thirsk, designed the plans.

